# Supplementary figures and images for: Cellular labeling of endogenous retrovirus replication (CLEVR) reveals de novo insertions of the gypsy retrotransposable element in cell culture and in both neurons and glial cells of aging fruit flies
Source: PLoS Biol. 2019 May 16;17(5):e3000278. doi: 10.1371/journal.pbio.3000278 (PMC6541305; doi:10.1371/journal.pbio.3000278)

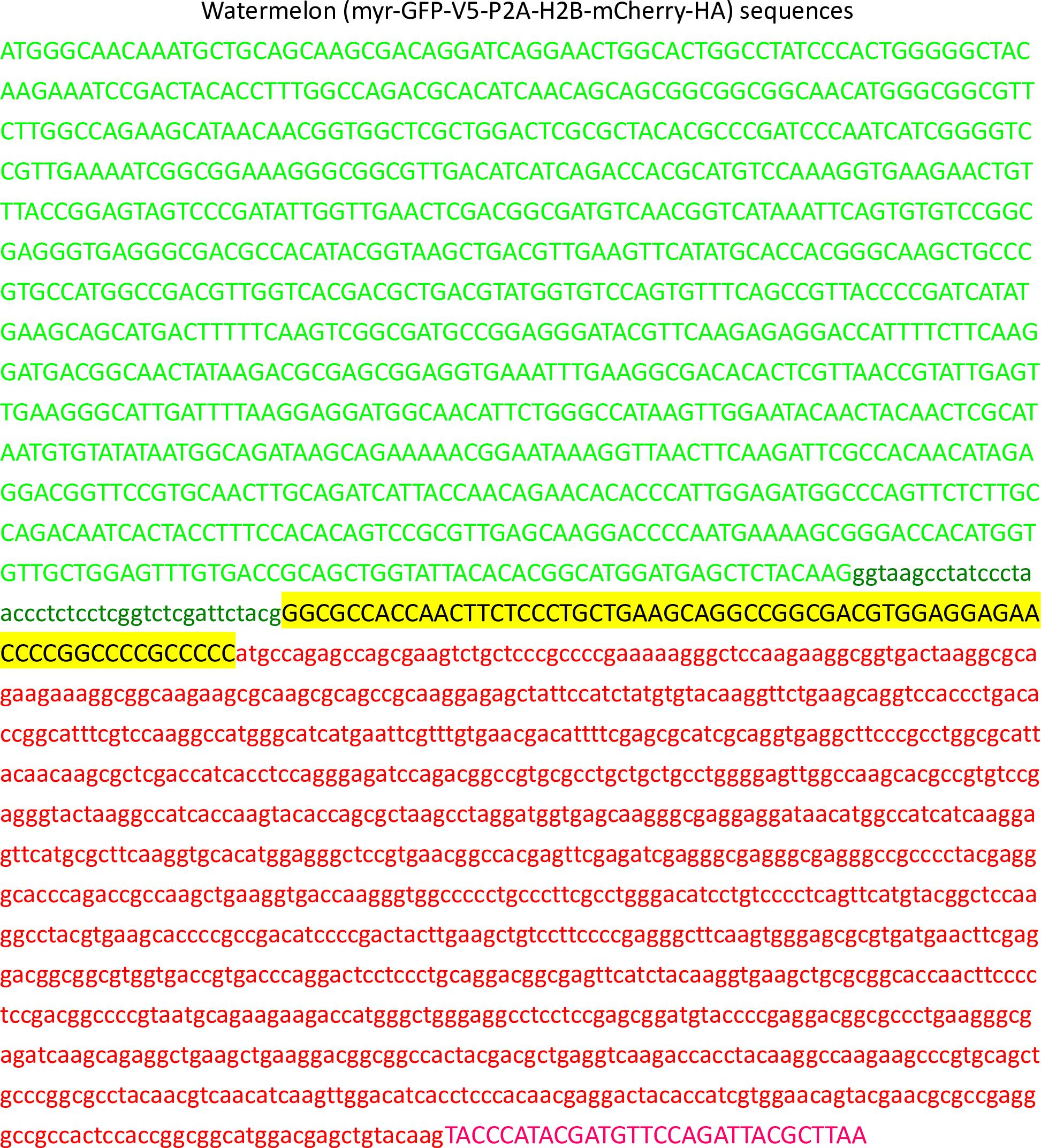

Supplement: S1 Fig — Sequence of WM (myr-GFP-V5-P2A-H2B-mCherry-HA). Myr-GFP sequence was shown in green, with the V5 tag in dark green. P2A sequence is shaded with yellow. H2B-mCherry is shaded red, with HA tag in pink. Myr-GFP, myristylated-GFP; P2A, porcine teschovirus-1 2A; WM, Watermelon. (TIF) [file pbio.3000278.s001.tif]

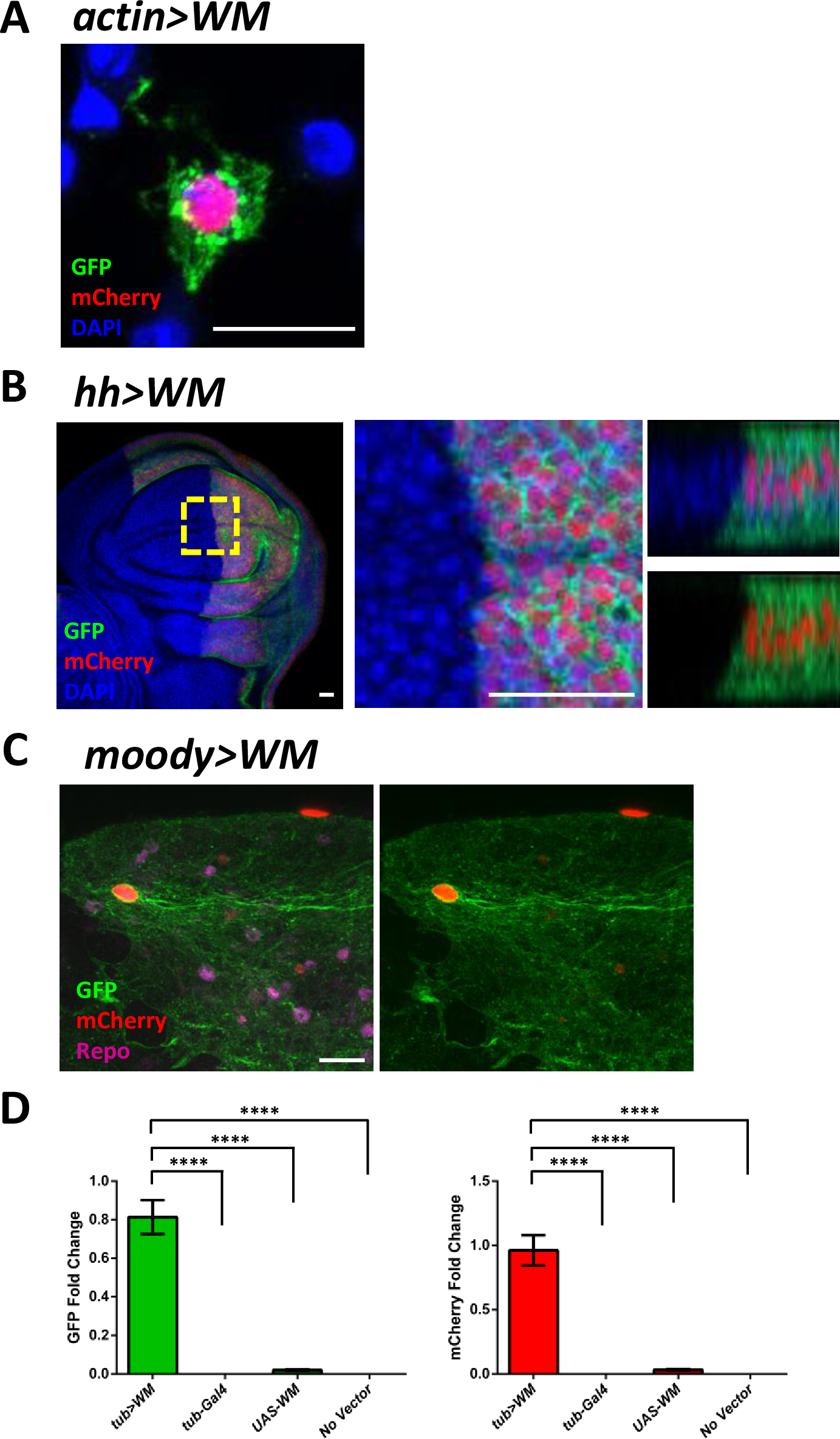

Supplement: S2 Fig — (A) Drosophila S2 cells were co-transfected with actin-Gal4 and UAS-WM (myr-GFP in green, H2B-mCherry in red, and DAPI in blue). Scale bar = 10 μm. (B) Wing discs were dissected from flies, with hh-Gal4 driving UAS-WM (hh>WM) at the third instar stage (myr-GFP in green, H2B-mCherry in red and DAPI in blue). Posterior cells expressing WM within the yellow dashed box are shown in high magnification. A 3D image is shown from Z series sections of the wing disc. Scale bar = 20 μm. (C) Moody Gal4 driving UAS-WM in SPG (moody>WM). Brains were dissected from adult flies and stained with glial marker Repo (magenta). Scale bar = 20 μm. (D) An RT-qPCR approach is used to confirm the functionality of GFP and mCherry primer sets. Each value from these experimental groups was further normalized to the tub>WM group (tub-Gal4 and UAS-WM co-transfection) in order to get the relative fold change. The relative fold change of both GFP (tub>WM: 0.81 ± 0.09, tub-Gal4: undetectable, UAS-WM: 0.02 ± 0.00, No vector: undetectable) and mCherry (tub>WM: 0.96 ± 0.12, tub-Gal4: undetectable, UAS-WM: 0.03 ± 0.00, No vector: undetectable) are compared (data in S2 Data). n = 3 biological replicates (****p < 0.0001, unpaired t test). GFP, green fluorescent protein; hh, hedgehog; myr-GFP, myristilated-GFP; RT-qPCR, reverse-transcription quantitative PCR; SPG, subperineurial glia; tub, tubulin promoter; UAS, upstream activating sequence; WM, Watermelon. (TIF) [file pbio.3000278.s002.tif]

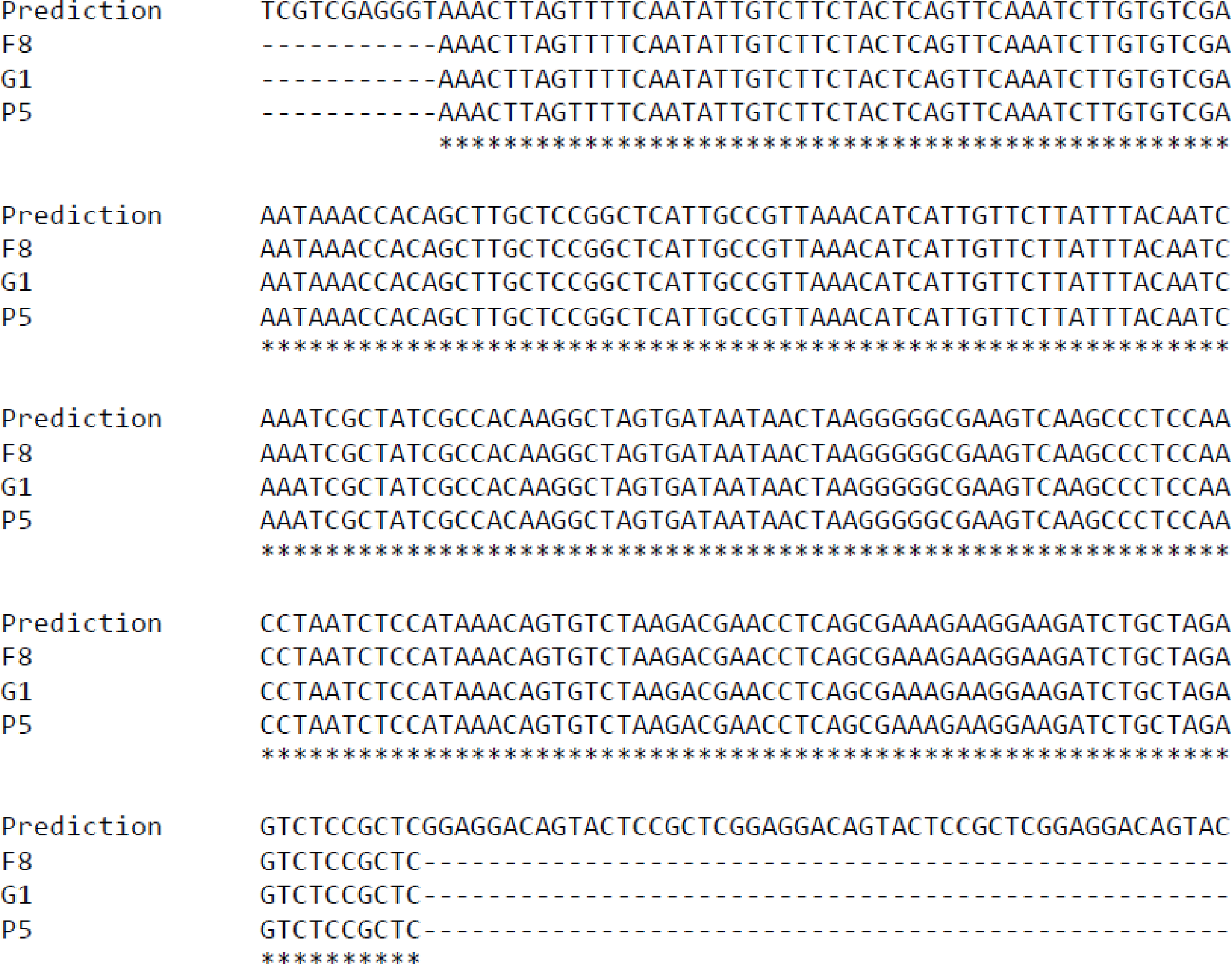

Supplement: S3 Fig — Sequencing comparison of nested PCR products from three different batches of aged gypsy-CLEVR flies, with comparison to the predicted sequence of gypsy-CLEVR rearrangement after retrotransposition. CLEVR, cellular labeling of endogenous retrovirus replication. (TIF) [file pbio.3000278.s003.tif]

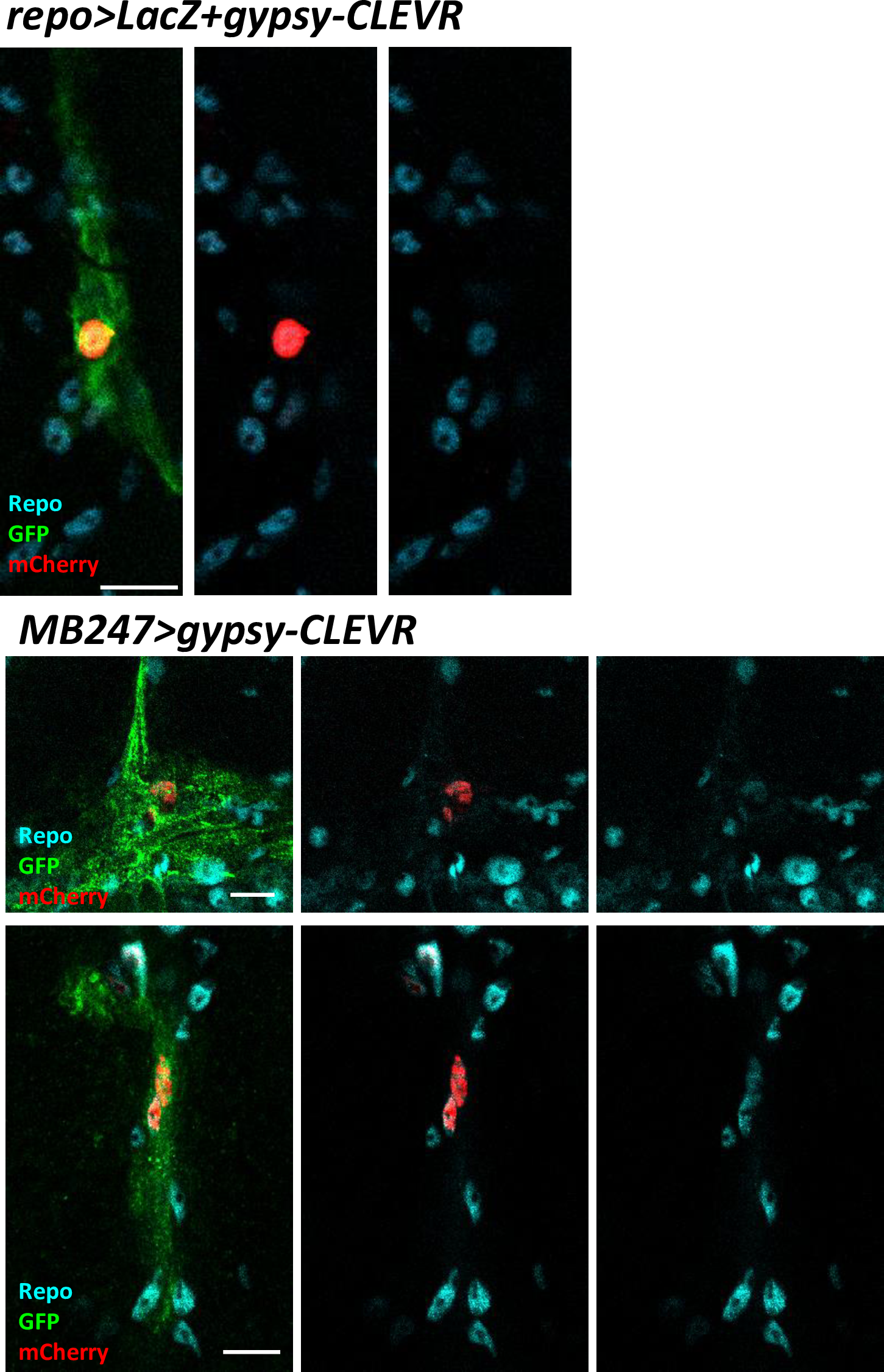

Supplement: S4 Fig — The gypsy-CLEVR was separately crossed with repo-Gal4 or MB247-Gal4. Adult brains from these crosses were dissected and labeled with glial marker (Repo in cyan) and both gypsy-CLEVR reporters, GFP (green, membrane) and mCherry (red, nuclei). Scale bar = 10 μm. CLEVR, cellular labeling of endogenous retrovirus replication; GFP, green fluorescent protein. (TIF) [file pbio.3000278.s004.tif]

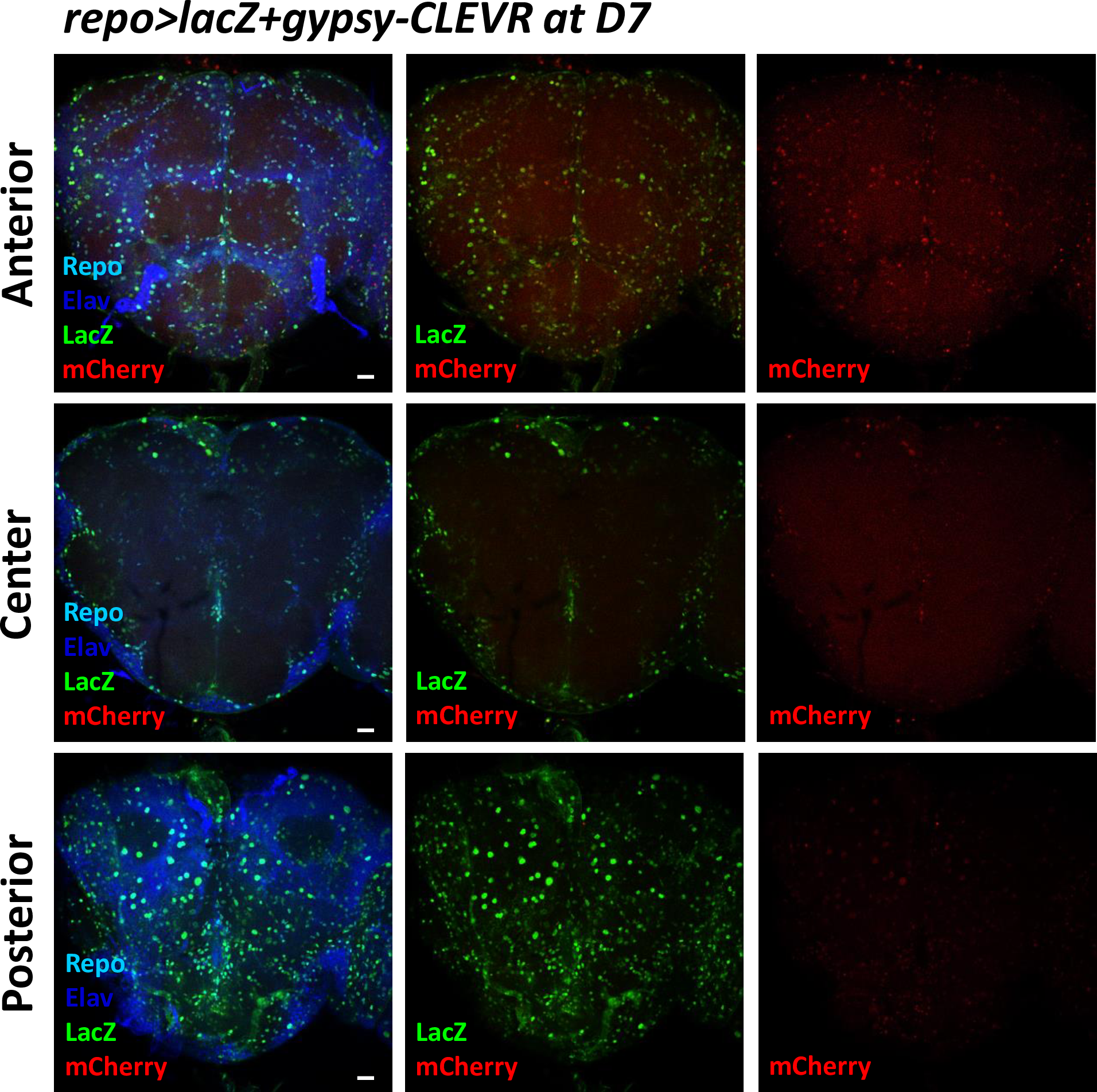

Supplement: S5 Fig — Optical sections of 7-day-old adult fly are shown from anterior, central, and posterior regions. Glial nuclei are labeled with the pan-glial marker Repo (cyan) and neuronal marker Elav (blue). Glial nuclei are independently labeled with UAS-nuclear-LacZ (green). gypsy-CLEVR reporter replication is revealed with nuclear mCherry (red). Highest levels of gypsy-CLEVR replication are seen in anterior sections. Scale bar = 20 μm. CLEVR, cellular labeling of endogenous retrovirus replication; UAS, upstream activating sequence. (TIF) [file pbio.3000278.s005.tif]

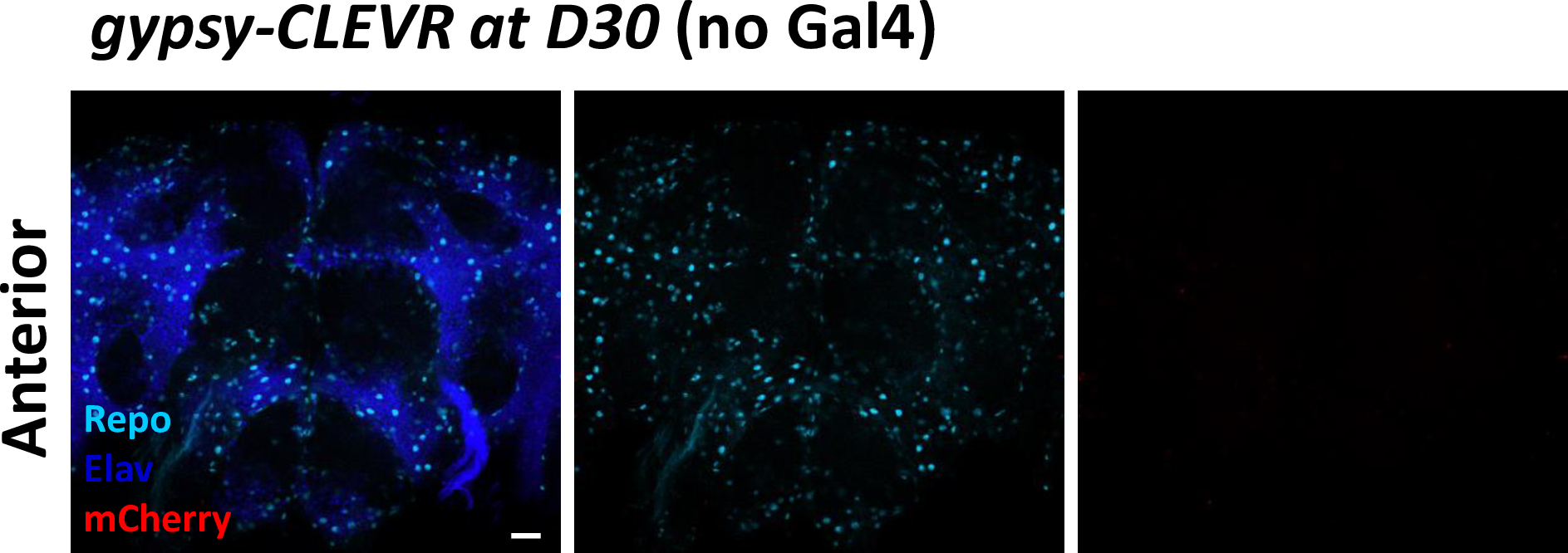

Supplement: S6 Fig — Gypsy-CLEVR transgenic flies that do not contain any Gal4 line were aged until 30 days, at which time high levels of gypsy expression and replication have taken place. Anterior brain sections of these aged gypsy-CLEVR adults contain few if any mCherry-labeled nuclei (red). Glial nuclei were counterstained with the Repo marker (cyan) and neuronal marker Elav (blue). Scale bar = 20 μm. CLEVR, cellular labeling of endogenous retrovirus replication; WM, Watermelon. (TIF) [file pbio.3000278.s006.tif]
